# Self-management by family caregivers to manage changes in the behavior and mood of their relative with dementia: an online focus group study

**DOI:** 10.1186/s12877-016-0268-4

**Published:** 2016-05-03

**Authors:** Judith Huis in het Veld, Renate Verkaik, Berno van Meijel, Paul-Jeroen Verkade, Wendy Werkman, Cees Hertogh, Anneke Francke

**Affiliations:** Department of Public and Occupational Health, EMGO Institute for Health and Care, Research, VU University Medical Center, Amsterdam, Netherlands; Netherlands Institute for Health Services Research (NIVEL), Utrecht, The Netherlands; Inholland University of Applied Sciences, Amsterdam, The Netherlands; Parnassia Psychiatric Institute, The Hague, The Netherlands; Department of Psychiatry, EMGO Institute for Health and Care Research, VU University, Medical Center, Amsterdam, The Netherlands; The Geriant Foundation, Region North of Amsterdam, The Netherlands; Dutch Alzheimer’s society, Amersfoort, The Netherlands; Department of General Practice and Elderly Care Medicine, EMGO+ Institute for Health and Care Research, VU Medical Center, Amsterdam, The Netherlands

**Keywords:** Self-management, Dementia, Changes in behavior and mood, Challenging behavior, Online focus groups, Internet discussion, Family, Informal caregivers

## Abstract

**Background:**

Self-management is important for family caregivers of people with dementia, especially when they face changes in their relative’s behavior and mood, such as depression, apathy, anxiety, agitation and aggression. The aim of this study is to give insight into why these changes in behavior and mood are stressful for family caregivers, what self-management strategies family caregivers use when managing these changes and the stress they experience.

**Methods:**

A qualitative study was conducted using four online focus groups with 32 family caregivers of people with dementia living in the Netherlands. Transcripts of the focus group discussions were analyzed using principles of thematic analysis.

**Results:**

Managing changes in the behavior and mood of their relative with dementia is stressful for family caregivers because of constantly having to switch, continuously having to keep the person with dementia occupied and distracted, the fact that others see a different side to the relative, and the fact that caregivers know what to do, but are often not able to put this into practice. Caregivers use calming down and stimulation as self-management strategies for influencing the changes in the behavior and mood of their relative. Furthermore, caregivers describe three self-management strategies that let them manage their own stress and keep up the care for their loved ones: looking for distractions, getting rest, and discussing their feelings and experiences.

**Conclusions:**

Behavior and mood changes of a person with dementia are stressful for family caregivers. They use several self-management strategies to positively affect the mood and behavior changes, and also to manage their own stress.

**Electronic supplementary material:**

The online version of this article (doi:10.1186/s12877-016-0268-4) contains supplementary material, which is available to authorized users.

## Background

Changes in behavior and mood are common in people with dementia. Approximately 90 % of people with dementia experience behavioral and mood changes in the course of their disease [1]. These changes concern symptoms (or clusters of symptoms), such as depression, apathy, agitation and aggression [2, 3]. Changes in behavior and mood are prompted in part by the interaction between the person with dementia and their family caregivers [4, 5]. This makes managing changes in behavior and mood a challenge for family caregivers, in all phases of the dementia of their relative [6].

The term ‘self-management’ is widely used these days by experts and professionals when talking about managing the impact of a disease in daily life. Following the definition of the national Dutch care standard self-management [7], which is largely based on the well-known definition of Barlow et al. [8], we define self-management as managing the chronic condition (symptoms, treatment, physical and psychological and social consequences, and related changes in lifestyle) so that the condition is optimally incorporated into daily life. Self-management is important not only for the patient but also for family caregivers [9]. Because dementia is a progressive condition, the patient becomes increasingly dependent on the family caregiver. This is partly why family caregivers have to make a significant contribution to the self-management of mood and behavioral changes of their relative with dementia. Furthermore, caregivers also have self-management tasks in managing their own stress resulting from, for example, managing the depressed mood or aggressive behavior of their relative with dementia [10].

A limited number of previous studies also described ways in which family caregivers manage with changes in the behavior and mood of their relative with dementia in their daily life [11–13]. These studies use the terms “management strategies”, “strategies” or “types of approach”. In a previous study from The Netherlands, The Vugt et al. found that nurturing and supporting were the most frequent strategies used, when family caregivers were faced with behavior or mood changes of their relative with dementia [11]. In Australian research, Moore et al. described various strategies that family caregivers use in response to behavioral and psychological symptoms of dementia: encouraging activities, utilizing psychotropic medications, identifying triggers, restraining or treating in a paternalistic manner, and meeting physiological needs, were the most commonly used strategies [12]. Furthermore, Turner et al. described strategies as well, namely in a study among family caregivers with a Latino background living in the USA. These caregivers tried to manage challenging behaviors of their relative with dementia, by acceptance, love, patience, adaptability, and establishing routines of care [13]. The afore mentioned studies are scarce examples of research on family carers’ strategies to deal with behavioral and mood changes in a relative with dementia [11–13].

Even less research has been done on the self-management strategies family caregivers use for managing the stress they themselves experience when faced with behavioral and mood changes in a relative with dementia. One of the rare studies in this field is done by Grigorovich et al. among adult sons caring for a parent with dementia in Canada. Strategies used to manage their stress included boundary setting and practicing self-care [14]. In addition, an integrated literature review of Caceres et al. described that the most frequently used strategies to manage caregiver stress in dementia, concerned adaptation and reframing. Caceres et al. also concluded that strategies to reduce family caregiver stress are poorly understood [15].

The aim of this paper is to give more insight into the stress that family caregivers experience when there are changes in behavior and mood of their relative with dementia, and into the self-management strategies of family caregivers for managing those changes and for managing their own stress. Such insights will be helpful for nurses, casemanagers and other health care professionals in supporting family caregivers’ self-management.

The specific research questions are:

1. Which aspects do family caregivers experience as stressful when they are faced with changes in behavior or mood (such as agitation, restlessness, apathy, and aggression, depression and anxiety) in their relative with dementia?
2. What self-management strategies do family caregivers use to manage these changes in the behavior and mood of their relative?
3. What self-management strategies do family caregivers use to manage their own stress when faced with behavioral and mood changes of their relative?

## Methods

### Design

We ran online qualitative focus group discussions with family caregivers of people with dementia. An online focus group involves using a secure website on the Internet to conduct group discussions [16, 17]. The online variant of the focus group was chosen as this made it possible to reach family caregivers who would not easily be able to travel because of the commitment of caring for their relative with dementia.

### Sample and recruitment

We recruited participants from an existing nationwide panel of family caregivers that is regularly used by the Dutch Alzheimer’s society (http://www.alzheimerpanel.nl/). A total of 240 family caregivers were selected at random from the panel (*n* = 1200). These caregivers were sent an e-mail from the Dutch Alzheimer’s society, inviting them to take part in an online focus group if they met the specified criteria for inclusion. The following inclusion criteria applied:

- The caregiver had to be a relative of a person diagnosed with dementia.
- The caregiver had to have contact with the person with dementia at least once a week.
- The person with dementia had to live at home (not in a care institution).
- The family caregiver had to have access to the Internet on a daily basis during the online focus group period.
- The family caregiver had to be aged at least 18.

In total, 37 family caregivers sent an e-mail expressing that they met the specified criteria for inclusion and that they were willing to participate. These family caregivers were sent an information letter by post with a form for giving consent. A total of 36 family caregivers returned the consent form saying that they wanted to take part. Of this group, 32 family caregivers actually participated in an online focus group by posting comments on the website. These 32 individuals are therefore considered to be the study participants.

### Online focus groups

There were four online focus groups in total between October 2014 and March 2015. Each focus group had seven to ten participants. Prior studies showed that this is a good number of participants to have for online discussions [18]. Only people who received a personal login code from the lead researcher (JH) could access this secure website. Participation was anonymous.

Over a period of two weeks, participants could log in to the secure website 24 h a day. Every second day, with the exception of weekends, one of the researchers (JH) added a new question (see below). Two of the authors (JH and RV) led the discussion by posing questions and summarizing reactions. They also send e-mail messages to participants who had not yet responded, if needed.

The following topics and questions were – amongst others - addressed in the online focus groups and form the basis of this article:

- Dementia often goes hand in hand with changes in the behavior or mood, such as irritability, restlessness, lack of initiative, aggressive behavior, depression, and anxiety. Do you recognize these changes in behavior and mood?
- How do you respond to changes in the behavior and mood of your relative with dementia?
- What effect does your way of responding to changes in the behavior and mood of your relative with dementia have?
- As a family caregiver, what is important in enabling you to manage these changes in your daily life?

### Data analysis

The data collection and analysis was an iterative process, following thematic analysis principles [19], and ultimately leading to data saturation.

The analysis started with familiarization with data, through reading and rereading the transcripts of the online focus groups. Following that, relevant excerpts within the transcripts were marked and tagged with keywords (codes). Initially, keywords were chosen that were close to the wording used by the participants. Then related codes were grouped as a way of identifying themes. After that, themes were named, and relationships between themes were studied and analyzed [19]. Main related themes are displayed in Additional file 1.

To improve the quality of the analyses and the trustworthiness, we used several strategies:

- The coding and ordering process of excerpts in the transcripts was supported by the MAXQDA11 software package.
- Triangulation of researchers was performed: all transcripts were first analyzed by two researchers independently: one trained as a nurse and health scientist (JH) and one trained as psychologist (RV). The coding and the interpretation of the codes were then discussed by these two researchers to deepen their analyses and to reach consensus about what were main themes. In addition, the other authors (BM, PJV, WW, CH & AF) each read and analyzed at least one transcript.
- All authors commented on interim and final analyses of the online focus group discussions. The authors have various educational backgrounds (nursing, health sciences, medicine, ethics, psychology, sociology) and various professions (researcher, professor, casemanager dementia, staff member of Alzheimer association). Furthermore, some authors have personal experiences as family caregiver of a person with dementia, which was also important for the quality of the analyses and for the trustworthiness of the results.

The qualitative methods and reporting of results adhere to the COREQ (Consolidated Criteria for Reporting Qualitative Studies) guidelines [20]. A complete COREQ checklist has been uploaded in Additional file 2.

## Results

### Background characteristics

A total of 36 family caregivers signed up for an online focus group. Of these, 32 actually participated in the discussions in the online focus group. The group consisted of partners as well as children and children-in-law. The majority said that the first symptoms of dementia (usually Alzheimer’s disease) appeared two to five years ago (see Table 1).

**Table 1:** Background characteristics (*n* = 36)

Background characteristic	Number
Age family caregiver	
Average age (years): 61	
Range: 42–80	
Sex family caregiver	
Men	4
Women	32
Relationship of family caregiver with person with dementia	
Partner	17
Child or child-in-law	19
Highest educational attainment	
Primary school	3
High school (preparatory to vocational education) and vocational training	7
High school (preparatory to university education)	6
Professional or academic university	16
Missing	4
Type of dementia of the relative with dementia	
Alzheimer’s disease	14
Alzheimer’s disease with vascular components	3
Vascular dementia	1
Lewy body dementia	1
Frontotemporal dementia	1
Dementia (no further description)	16
First symptoms of dementia (according to the family caregiver)	
2–5 years ago	10
6–10 years ago	8
11–15 years ago	4
Not reported	14

All the family caregivers recognized changes in the behavior and mood of their relative with dementia, such as depression, anxiety, agitation, restlessness, or aggression. Managing these changes was stressful for them. Family caregivers mentioned several themes related to self-management. These are shown schematically in Additional file 1, and are elaborated below.

### Stressful aspects for the family caregiver

Changes in behavior and mood of the relative with dementia are stressful for family caregivers. Four stressful aspects are mentioned: (1) the continual switching; (2) continually keeping the relative with dementia occupied and diverted; (3) the fact that others see a different side to their relative; and (4) knowing what to do in theory, but often being unable to put it into practice.

#### Continual switching

Family caregivers indicate that the behavior of their relative can change during the day, for example from apathy to restlessness. This means that the family caregiver needs to keep switching between different self-management strategies, for example from a diversion strategy to a strategy involving acknowledgement. Continually switching is also needed to make the relative with dementia feel at ease.

*“I vary my responses. Sometimes I’m distracting her, sometimes acknowledging her feelings, sometimes choosing a different perspective, sometimes humbling myself and taking the blame. In other words: varied responses, depending on her needs.” (Respondent 28)*

#### Continually keeping the relative occupied and diverted

Family caregivers also indicate that it is hard to keep their relative occupied and diverted. The relative can be restless, for example if something is not in the usual place, if the family caregiver is doing something else, or if there are visitors. Keeping the person with dementia occupied and diverted is a real challenge. As a result, family caregivers may not have time for their own pursuits and hobbies. To cope with this, family caregivers “try to get over it” and look for the next source of distraction. However, they do not always manage this: “*I can’t do my hobby at home any longer. Our mentor told me to spend an hour in my hobby room whenever our household help is here, but unfortunately he keeps coming to ask questions. I miss my hobby an awful lot.” (Respondent 10)*

#### Others see a different side to the relative

It is also stressful to family caregivers that their relative can sometimes “put on a good show” in front of others. The people around them – not only friends and family but also healthcare professionals - do not see their relative like the family caregiver does. As a result, family caregivers can feel as if they have to defend themselves, because the real situation is different to how it appears to others. A partner said:

*“The biggest trap when dealing with people with dementia is that they can temporarily put on a good show. The relative is then told: “it’s not that bad” and* “*I didn’t notice anything,” etc.(…). It makes you feel like you constantly have to defend yourself and say they are not doing well at all.” (Respondent 1)*

Partners in particular indicate that others see a different side to their loved one. Partners have to manage with changes in behavior and mood on a daily basis.

#### Knowing how to respond in theory, but being unable to put in practice

Family caregivers often know what to do in theory, but in practice things can be quite different. This can happen because the situation always develops in a different way to what was expected, or because it is difficult to accept the situation. Being able to accept a situation or not influences how you respond to your relative with dementia. A daughter said: *“It is very hard for me to handle her behavior. (…) I know that you shouldn’t mention it and/or joke about it. But I’ve noticed that I still do it. I’m still having a hard time accepting how much my mother has changed (…).” (Respondent 24).* Partners, children, and children-in-law all indicate that they learn every day how to respond better to changes in the behavior and mood of their relative with dementia. Family caregivers often read up about dementia, and they learn from their daily interactions with their relative. Sometimes family caregivers have experience themselves working in professional healthcare. However, theory is different from practice.

### Self-management strategies of family caregiver to manage the changes in behavior and mood

Family caregivers use various self-management strategies to cope adequately with changes in the behavior and mood of their relative with dementia in their daily lives. In the analyses, these strategies were grouped into two themes: calming down and stimulation.

#### Calming down

Calming down involves for example remaining tranquil, being patient, and adapting to the mood state of the relative with dementia. Family caregivers say that they remain calm and keep their patience on those occasions when the relative with dementia is anxious or aggressive. These are self-management strategies intended to prevent the situation from getting out of hand, for example aimed at avoiding their relative becoming aggressive by exercising caution in their contact with their relative. Family caregivers also try to adapt to their relative’s mood state in order to reduce tension or restlessness in their relative.

#### Stimulation

Family caregivers also mention stimulation as a self-management strategy. This includes telling stories, for example, humor, being positive, and encouraging activities and distractions. Family caregivers tell positive stories and try to be upbeat in an effort to haul their relative out of a negative spiral, for example in the case of depression or apathy, or help improve their mood. Family caregivers also mention encouraging activities and distractions such as getting out of the house for a bit or a trip to the shops. In distracting their relative, family caregivers are trying to make sure that the person with dementia does not become more restless or suspicious. A daughter-in-law explained: *“I try to change something in the situation, so that a new door opens in his head or an old one closes. For example by getting out of the house with him. Even if we just walk up and down a couple of streets on the sidewalk, or go into the garden and back. To see if we can find a different subject while we’re out to keep his mind occupied. (…) But it’s more likely that I end up looking for the next distraction. Off to the shops. Etc.” (Respondent 35)*

### Self-management strategies to manage the own stress

Self-management also means that the family caregiver needs to find a satisfactory way of managing the stress they experience in their daily lives from managing their relative’s changes in behavior and mood. The self-management strategies adopted by family caregivers are: looking for distractions, getting rest, and discussing their feelings and experiences.

#### Looking for distractions

Looking for distractions is a strategy for managing changes in behavior and mood on a daily basis. Family caregivers deliberately plan activities for themselves, such as pursuing hobbies, meeting up with family and friends, or going on holiday. “*I definitely find caring for a mother with dementia who lives on her own hard and very stressful for everyone in my own family and my sister’s family. It takes up a lot of time and energy that would otherwise be spent on my family and my social life. (…) We arrange to do (…) fun things with friends so that I can relax at least 1 day/evening and we plan short breaks a bit more often. I do explicitly make plans for relaxation.” (Respondent 9).*

#### Getting rest

In addition to looking for distractions, getting rest is another strategy for managing the stress and being able to keep up the care. Family caregivers need to recharge their batteries in order to be able to carry on helping their relative during changes in behavior and mood.

*“I make sure I get enough rest, so I don’t keep rushing around if it’s getting too much for me, in order to be able to cope properly with these changes. My husband can’t walk anymore so he often sleeps in his wheelchair. Then I have a lie-down too and relax with some music.” (Respondent 4).*

#### Discussing feelings and experiences

Talking to friends and family about the changes in the relative’s behavior and moods is another strategy that family caregivers use in order to be able to keep up the care. Discussing it with care professionals such as a case manager can also be a strategy:

*“I often find my partner behaving irritably, which he never did before (…). He is more friendly to the home care people than he is to me, which is why I also find it difficult to be nice to him. Am I jealous? This is something I find I can discuss with my case manager.” (Respondent 4)*

## Discussion

Family caregivers experience stress from the changes in the behavior and mood of their relative with dementia, such as agitation, restlessness, apathy, aggression, depression, and anxiety. They find it stressful because they are continually switching, because they are continually having to keep the relative with dementia occupied and diverted, because others see a different side to their relative, and because family caregivers know what to do in theory, but are often unable to put this into practice. From previous research we do know that caregiver stress resulting from changes in the behavior or mood of the person with dementia is a very frequent problem, and exists in the initial stages as well as in subsequent stages of dementia [6]. This underlines the necessity to support family caregivers in their self-management of the changes in behavior and mood, and also in managing their own stress.

Keeping calm and stimulation are self-management strategies that family caregivers apply to influence changes in the behavior and mood of their relative. Calming down involves for example remaining tranquil, being reassuring, and adapting to the mood state. Stimulation can involve telling stories, encouraging activities, and providing distraction.

Self-management strategies that family caregivers use to manage their own stress in their daily lives and to keep up their care for their relative when there are behavioral and mood changes are looking for distractions, getting rest, and discussing feelings and experiences.

Calming down and stimulation by family caregivers in managing changes in the behavior of people with dementia have been mentioned in previous studies [11–13]. What our study adds is that family caregivers often know which strategies would be worthwhile but they are not always able to put this into practice. For example because the situation does not develop as they expected or because it is difficult to accept the situation. They can also experience stress from always having the person with dementia near them and from the fact that other people see a different side to the relative. As a result, they do not always manage to deal appropriately with the changes in behavior and mood. Quinn et al. point out that family caregivers need to find a balance between giving the person with dementia the best care they can and caring for themselves [21]. Our study shows that looking for distractions, getting rest, and discussing feelings and experiences are important self-management strategies for family caregivers that enable them to manage the stress.

### Practical recommendations

An understanding of what family caregivers find stressful and what self-management strategies family caregivers adopt can be used when giving shape to self-management support interventions. Self-management support can for example be provided by nursing staff and dementia case managers. This study showed that family caregivers experience a great deal of stress from the changes in the behavior and mood of their relative with dementia. Family caregivers find it stressful that other people do not believe them. Nursing staff can take note of this by listening to the family caregiver and asking them about the situations in which they see behavioral or mood changes.

Family caregivers also experience stress because, even when they do know how they should act, they are not always able to put this into practice. Despite the information they obtain from books, their day-to-day experiences and in some cases their professional knowledge, knowledge alone often turns out not to be enough. In addition to providing information, nursing staff can also support family caregivers in developing the skills that let them respond to changes in behavior and mood.

Professionals, such as nursing staff and dementia case managers need to be aware of the supportive needs of family caregivers. Talking about feelings and experiences is an important self-management strategy for managing the stress and being able to keep up the family care. Nursing staff and case managers can take this into account by explicitly inquiring into the feelings and experiences of family caregivers when faced with changes in the behavior and mood of their relative with dementia. In addition, giving information about how to manage with behavioral and mood changes is important, as well as informing family caregivers about the opportunities of support groups of family caregivers appears to be important: a recent systematic meta-review showed that evidence exists for support groups, which were shown to relieve stress of family caregivers [9]. Evidence was also found for self-management support interventions that increased family caregivers’ knowledge about how to deal with problems, such as behavioral and mood changes [9].

### Limitations and strengths of the study

A strength of this study is that we used several strategies to improve the quality of analysis and trustworthiness of results: coding and ordering through support of software package for qualitative analysis, triangulation of researchers, and discussions of interim and final analyses with authors with different backgrounds, among which also authors who have personal experiences with family care for a person with dementia. However, a limitation of this study is that most of the family caregivers had a high level of education. This will have consequences for the transferability of the results [22]. The study by de Vugt et al. showed that highly educated family caregivers adopt more often supportive care strategies and are better able to adjust to the functioning of the person with dementia than other family caregivers [11]. A second limitation that could affect the transferability is that the participating family caregivers were all members of an existent family caregiver panel, run by the Dutch Alzheimer’s society. This group may be more aware of developments and knowledge about dementia and possible self-management strategies for managing changes in behavior and mood than the average family caregiver.

Finally, a limitation is that we did not look at all the factors that cause stress. It is known that family caregivers can also find it difficult to manage other aspects of dementia, such as changes in the relationship or no longer being able to undertake shared activities [23]. While this article focuses on self-management by family caregivers in response to changes in behavior and mood, the daily stress that family caregivers have to manage with, is broader than just managing with these changes.

## Conclusions

For family caregivers changes in the behavior and mood of their relative with dementia, such as agitation, restlessness, apathy, aggression, depression, and anxiety, are stressful. The continual switching, keeping the relative with dementia occupied and distracted, the fact that others see a different side to their relative, and knowing what to do, but being unable to do so in practice, are particularly stressful.

The family caregivers of people with dementia use both calming down and stimulation as self-management strategies for managing changes in the behavior and mood of their relative. They also describe self-management strategies (looking for distractions, getting rest, and discussing their feelings) for keeping up the day-to-day care in the face of these changes.

An understanding of self-management by family caregivers when managing changes in the behavior and mood of their relative can help professionals to provide suitable support to family caregivers.

### Ethics approval and consent to participate

The study protocol was approved by the Medical Ethics Committee of the VU University Medical Center (reference 2014.323). This committee had no objections to the study. All participants received written information about the purpose and method of the online focus groups and signed an informed consent form prior to participation in the online focus groups.

### Consent for publication

Not applicable.

### Availability of data and materials

The Dutch language transcripts of the online focus groups are stored in anonymized form in NIVEL - Netherlands Institute for Health Services Research. Persons who are interested in these transcripts, can contact a.francke@nivel.nl.

## Additional files

Additional file 1: Stressful aspects and self-management strategies by family caregivers when there are changes in their relative’s behavior and mood. (DOC 36 kb) Additional file 2: Consolidated criteria for reporting qualitative studies (COREQ): 32-item checklist. (DOC 66 kb)
