# Supplementary material for: Self-management by family caregivers to manage changes in the behavior and mood of their relative with dementia: an online focus group study
Source: BMC Geriatr. 2016 May 3;16:95. doi: 10.1186/s12877-016-0268-4 (PMC4855870; doi:10.1186/s12877-016-0268-4)
Supplement: Additional file 1: — Stressful aspects and self-management strategies by family caregivers when there are changes in their relative’s behavior and mood. (DOC 36 kb) [file 12877_2016_268_MOESM1_ESM.doc]

**ADDITIONAL FILE 1. *Stressful aspects and self-management strategies by family caregivers when there are changes in their relative’s behavior and mood***

**Self-management strategies of family caregiver to manage changes in behavior or mood:**

- Calming down

- Stimulation

**Stressful aspects for family caregiver:**

- Continually switching
- Continually keeping the relative occupied and diverted
- Others see a different side
- Knowing how to respond in theory, but not in practice

**Self-management strategies of family caregiver to manage own stress:**

- Looking for distractions
- Getting rest
- Discussing feelings and experiences

**Changes in behavior or mood of person with dementia**
