# Supplementary material for: Self-management by family caregivers to manage changes in the behavior and mood of their relative with dementia: an online focus group study
Source: BMC Geriatr. 2016 May 3;16:95. doi: 10.1186/s12877-016-0268-4 (PMC4855870; doi:10.1186/s12877-016-0268-4)
Supplement: Additional file 2: — Consolidated criteria for reporting qualitative studies (COREQ): 32-item checklist. (DOC 66 kb) [file 12877_2016_268_MOESM2_ESM.doc]

**ADDITIONAL FILE 2.** Consolidated criteria for reporting qualitative studies (COREQ): 32-item checklist

**Self-management by family caregivers to manage changes in the behavior and mood of their relative with dementia: an online focus group study**

Judith Huis in het Veld; Renate Verkaik; Berno van Meijel; Paul-Jeroen Verkade; Wendy Werkman; Cees Hertogh; Anneke Francke

| **NO. and item** | **Guide questions/ description** | **Response** |
| --- | --- | --- |
| **Domain 1: Research team and reflexivity** |  |  |
| **Personal Characteristics** |  |  |
| 1. Interviewer/ facilitator | Which author/s conducted the interview or focus group? | *Judith Huis in het Veld (JH) and Renate Verkaik (RV)* |
| 2. Credentials | What were the researcher's credentials? | *JH: RN and MSc*  *RV: PhD* |
| 3. Occupation | What was their occupation at the time of the study? | *Both were researchers* |
| 4. Gender | Was the researcher male or female? | *Female* |
| 5. Experience and training | What experience or training did the researcher have? | *JH: Nursing and Health Sciences ; RV : psychology* |
| **Relationship with participants** |  |  |
| 6. Relationship established | Was a relationship established prior to study commencement? | *No* |
| 7. Participant knowledge of the interviewer | What did the participants know about the researcher? | *They knew only the* reasons for doing the research and the affiliations of the researchers |
| 8. Interviewer characteristics | What characteristics were reported about the interviewer/facilitator? | *R*easons and interests in the research topic |
| **Domain 2: study design** |  |  |
| **Theoretical framework** |  |  |
| 9. Methodological orientation and Theory | What methodological orientation was stated to underpin the study? | *Thematic* analysis |
| **Participant selection** |  |  |
| 10. Sampling | How were participants selected? | Purposive sampling |
| 11. Method of approach | How were participants approached? | *By* email |
| 12. Sample size | How many participants were in the study? | *37 were interested to participate, and received the information letter* |
| 13. Non-participation | How many people refused to participate or dropped out? | *4 did not start or dropped out, for unknown reasons* |
| **Setting** |  |  |
| 14. Setting of data collection | Where was the data collected? | *Online discussion group* |
| 15. Presence of non-participants | Was anyone else present besides the participants and researchers? | *No* |
| 16. Description of sample | What are the important characteristics of the sample? | *Majority were female, and partner or adult child of a person with dementia* |
| **Data collection** |  |  |
| 17. Interview guide | Were questions, prompts, guides provided by the authors?  Was it pilot tested? | *Yes an interview guide with semi structured questions was used*.  *The content of the draft interview guide was discussed with the project group, in which also persons were involved who were relatives of a person with dementia themselves.* |
| 18. Repeat interviews | Were repeat interviews carried out? | *In the online focus group strategy used, the discussion site was open for a period of two weeks. Within that period questions were posed every two or three days.* |
| 19. Audio/visual recording | Did the research use audio or visual recording to collect the data? | *Not applicable; in online focus groups the participants themselves type their reactions. The reactions are automatically transferred to a transcript ready for analyses.* |
| 20. Field notes | Were field notes made during and/or after the interview or focus group? | *Not applicable for online focus groups.* |
| 21. Duration | What was the duration of the interviews or focus group? | *In each online focus group, the discussion site was open for a period of two weeks.* |
| 22. Data saturation | Was data saturation discussed? | *Yes* |
| 23. Transcripts returned | Were transcripts returned to participants for comment and/or correction? | *No, not applicable, because the participant him/herself typed their own reactions* |
| **Domain 3: analysis and findings** |  |  |
| **Data analysis** |  |  |
| 24. Number of data coders | How many data coders coded the data? | *Two, JH and RV.* |
| 25. Description of the coding tree | Did authors provide a description of the coding tree? | *Yes.* |
| 26. Derivation of themes | Were themes identified in advance or derived from the data? | *Derived form the data* |
| 27. Software | What software, if applicable, was used to manage the data? | *Maxqda11* |
| 28. Participant checking | Did participants provide feedback on the findings? | *No* |
| Reporting |  |  |
| 29. Quotations presented | Were participant quotations presented to illustrate the themes / findings?  Was each quotation identified? | *Yes* |
| 30. Data and findings consistent | Was there consistency between the data presented and the findings? | *Yes* |
| 31. Clarity of major themes | Were major themes clearly presented in the findings? | *Yes* |
| 32. Clarity of minor themes | Is there a description of diverse cases or discussion of minor themes? | *Yes* |

**Reference**:

20. Tong A, Sainsbury P, Craig J. Consolidated criteria for reporting qualitative research (COREQ): a 32-item checklist for interviews and focus groups. *International Journal for Quality in Health Care*. 2007. Volume 19, Number 6: pp. 349 – 357
